# Development and Validation of the B Cell-Associated Fc Receptor-like Molecule-Based Prognostic Signature in Skin Cutaneous Melanoma

**DOI:** 10.1155/2020/8509805

**Published:** 2020-08-22

**Authors:** Yu Liu, Yiding Chen, Xianyu Hu, Jialin Meng, Xiaojing Li

**Affiliations:** ^1^Department of Plastic Surgery, First Affiliated Hospital of Anhui Medical University, Hefei, Anhui, China; ^2^Department of Urology, The First Affiliated Hospital of Anhui Medical University; Institute of Urology & Anhui Province Key Laboratory of Genitourinary Diseases, Anhui Medical University, Hefei, Anhui, China; ^3^Department of General Surgery, The First Affiliated Hospital of Anhui Medical University, Hefei, Anhui, China

## Abstract

**Methods:**

461 patients with CM from The Cancer Genome Atlast- (TCGA-) CM cohort and 290 pateints from the GSE65904 cohort were enrolled. Student's *t*-test was used to compare the differences, and Pearson's correlation coefficient was employed to evaluate associations. The Kaplan-Meier (K-M) survival analysis was used to evaluate overall survival (OS). The multivariate Cox regression was conducted to generate the FCRL prognostic signature. GSEA analysis and TIMER were employed to study the potential mechanisms.

**Result:**

Patients with Breslow's depth high or equal to 3 cm had the lower expression of FCRL1-6 (all, *P* < 0.05), which indicates poor OS, as well as age, stage, and Breslow's depth subgroups (all, *P* < 0.001). The overall FCRL1-6 prognostic signature was generated in the TCGA cohort (K-M, *P* < 0.001; area under the curve (AUC), 0.649 for 3-year OS) and validated in the GSE65904 cohort (K-M, *P* < 0.001; AUC, 0.659 for 3-year OS). The GSEA results revealed that high expression of FCRLs indicated activated immune-associated pathways, and FCRLs are positively associated with the infiltration of B cells.

**Conclusion:**

Highly expressed FCRLs were observed associated with a favourable OS of CM. FCRL1-6-based prediction signature could act as a biomarker to predict the prognosis of patients with CM.

## 1. Introduction

Cutaneous melanoma (CM) causes the highest absolute number of deaths among all skin tumors and consists of four main types: lentigo malignant melanoma, nodular melanoma, acral lentiginous melanoma, and superficial spreading malignant melanoma [1, 2]. Globally, melanoma accounted for 1.6% of new cancer cases and caused 0.6% of cancer-specific deaths in 2018 [3]. Furthermore, the mortality of CM has increased sharply in the past decades: The median mortality of CM was ~1.55 per 10,000 individuals for males in 1985-1987 and increased to 2.57 in 2013-2015, while the median mortality of females also increased from 1.39 to 1.55 [4]. In the USA, there were ~1,000,000 new cases and 7,000 CM-related death in 2019, and both of them are higher in males compared with those in females [5]. Several factors have been reported to affect the risk of CM; among them, ultraviolet (UV) light plays a key role [6], especially indoor solar UV exposure, which leads to high UV exposure and depletion of vitamin D3. Sunburn, cosmetic ingredients, sex hormones, family history, and DNA mutations are all risk factors for CM [7].

The association between defects in the immune system activity and CM has been evaluated by several studies, which demonstrated that alterations in immune cells and proteins are potential factors for immunosurveillance and progression of CM [8–10]. Park et al. showed that tissue-resident memory CD8+ T cells could promote a durable melanoma-immune equilibrium that is confined to the epidermal layer of the skin [11]. Eggermont et al. reported that ipilimumab, at a dose of 10 mg per kg of body weight, resulted in significantly higher rates of recurrence-free survival, overall survival (OS), and distant metastasis-free survival than those of the placebo in high-risk Stage III melanoma [12]. Fc receptor- (FcR-) like (SRGRV) molecules belong to the immunoglobulin superfamily (IgSF) and have a potential activating and inhibitory role [13]. In humans, this family encodes six transmembrane receptors, FCRL1-6, and two cytoplasmic proteins, including FCRLA and FCRLB that lack any type of transmembrane region [14]. The expression profile of FCRLs has been widely investigated in several malignancies such as chronic lymphocytic leukemia (CLL) and acute lymphocytic leukemia (ALL) [15–17], in infectious diseases such as malaria, hepatitis, and acquired immunodeficiency syndrome [18–20], and in some autoimmune diseases [21–23]. It has been reported that FRCL gene expression may be upregulated in the progression of melanoma [24].

However, the mechanism of FCRLs in CM has not been yet elucidated. Therefore, the present study explored the different expression of FCRLs in subgroups of patients with CM and generated and validated the determined prognostic signature. Moreover, Gene Set Enrichment Analysis (GSEA) was employed to identify the key pathways among high-risk and low-risk groups. In addition, immune cell infiltration analysis was performed by the tumor immune microenvironment (TIME) online tool to deeply illustrate the association between FCRLs and the polarized prognosis of patients with CM.

## 2. Materials and Methods

### 2.1. Study Population

The Cancer Genome Atlas- (TCGA-) CM data were downloaded from UCSC Xena (https://tcga.xenahubs.net), including the Illumina HiSeq pan-cancer normalized gene expression data and the DNA methylation data from the HumanMethylation450 platform. An external validation cohort, GSE65904, was downloaded from the Gene Expression Omnibus database (https://www.ncbi.nlm.nih.gov/geo/).

### 2.2. Statistical Methods

Graphpad 8.0 was used to display the different expression of FCRLs in various subgroups of patients with CM. Student's *t*-test was used to compare the differences between unpaired values, while the Pearson's product-moment correlation coefficient was employed to evaluate the association between CpG sites and mRNA expression of FCRLs. The Kaplan-Meier (K-M) survival analysis of patient subgroups with high and low levels of FCRLs and clinicopathological characteristics was also performed.

### 2.3. Establishment and Validation of the Prognostic Signature

To evaluate the overall prognostic value of FCRLs, we generated the FCRLs prognostic signature based on the results of the multivariate Cox regression with the TCGA-CM cohort, and each patient obtained a risk score from the signature. The K-M survival analysis was used to indicate the different outcomes of risk groups with the “survminer” package. Next, the time-dependent receiver operating characteristic (ROC) curve analysis was used to calculate the area under the curve (AUC) for 1-year, 3-year, and 5-year OS and disease-free survival, and to determine the prediction accuracy of our model using the “survivalROC” package. The predicted value and accuracy were also validated in the GSE65904 cohort.

### 2.4. Gene Set Enrichment Analysis (GSEA) Analysis among FCRLs Determined Risk Groups

GSEA primarily analyzes microarray data, using genomic and genetic sequencing, to detect significant biological differences in microarray datasets [25]. In the present study, differentially expressed genes and common crucial pathways between FCRL-determined high- and low-risk groups were identified by GSEA. The datasets were normalized and the intensity of the log_10_ probe set was calculated using the Robust Multichip averaging algorithm with bioconductors [26]. The pathway analysis of each dataset was performed independently. The variability was measured in the interquartile range (IQR), and a cut-off was set to foreclose IQR values < 0.5 for all the remaining genes. If one gene was targeted in multiple probe sets, the probe set with the greatest variability was retained. Besides, genes in each pathway were subjected to the statistical analysis system (SAS), and each pathway's *P* value was obtained in the permutation test with ×1000. *P* < 0.05 was considered to indicate a statistically significant difference.

### 2.5. TIME Analysis by TIMER

TIME analysis was performed by the TIMER [27] online tool, aimed at deeply illustrating the association between FCRLs and the polarized prognosis of patients with CM. TIMER is a comprehensive resource for systematic analysis of immune infiltrates across diverse cancer types. The abundances of six immunocytes (B cells, CD4+ T cells, CD8+ T cells, neutrophils, macrophages, and dendritic cells) were estimated by the TIMER algorithm.

## 3. Results

### 3.1. Associations between mRNA Levels, DNA Methylation, and Pathological Features

**Figure 1:**
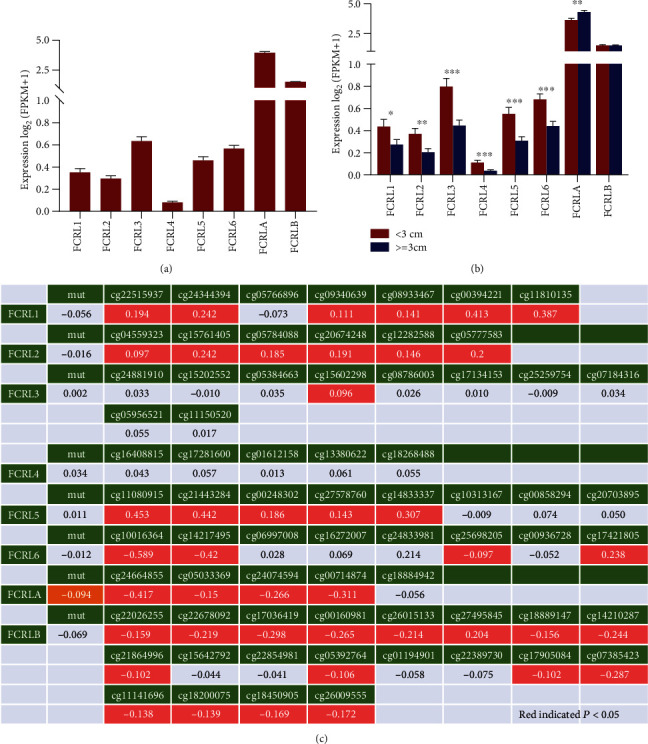
FCRLs expression in CM patients and the association with promoter DNA methylation. (a) The baseline of eight FCRLs in CM patients in the TCGA-CM cohort. (b) The different expression of FCRLs in patients with high (≥3 cm) or low (<3 cm) Breslow's depth. (c) The association of FCRLs and DNA methylation at their promoter region.

We firstly checked the baseline mRNA expression of all 8 FCRLs and found that FCRLA and FCRLB had a high level in all FCRLs. As for the analysis of the Breslow's depth, which indicates the depth of tumor cells have invaded the skin, we observed that patients with the Breslow's depth higher or equal to 3 cm had the lower expression of FCRL1-6 (all, *P* < 0.05), while FCRLA showed the opposite result (*P* < 0.05) and FCRLB showed no difference (Figure 1(b)). DNA methylation plays an important role as it affects the mRNA level of genes, so we evaluated the associations between DNA methylation and mRNA expression of FCRLs. We found that the cg11080915 had the most prominent effect on the mRNA level of FCRL5 among all the FCRLs, while cg10016364 had the lowest effect on FCRL6 (Figure 1(c)**)**.

### 3.2. The Prognostic Value of FCRLs and Pathological and Clinical Features

**Figure 2:**
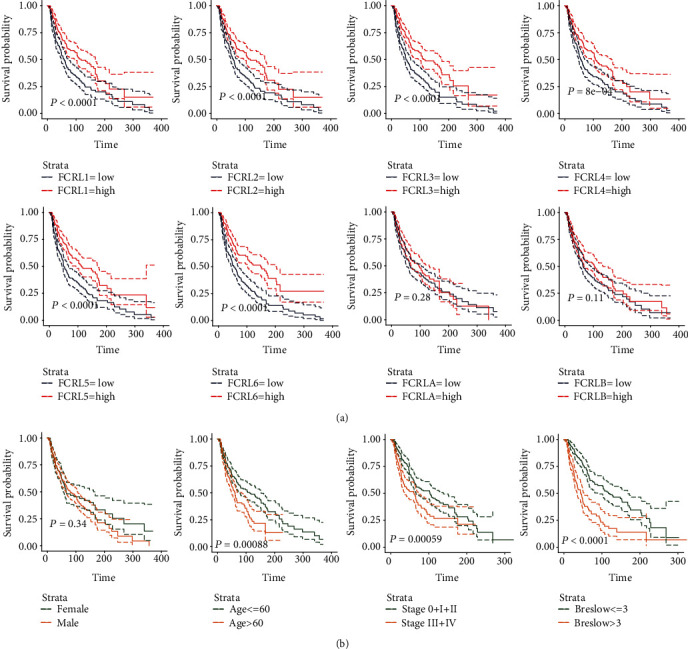
The K-M plot showing the different OS in subgroups. (a) Different OS outcomes in high- and low-expression groups of FCRLs. (b) Different OS outcomes in sex, age, stage, and Breslow's depth subgroups.

To assess the overall survival (OS) prognostic value of FCRLs, we divided the patients into two groups by the median of each FCRL expression. Interestingly, we found that the lower expression of FCRL1-6 depicted poor OS (all, *P* < 0.05, Figure 2(a)), which is consistent with the results shown in Figure 1(b). However, there are no significant results between FCRLA (*P* = 0.28) and FCRLB (*P* = 0.085) to CM OS (Figure 2(a)). What is more, we also evaluated the prognostic value of several pathological and clinical features. We exposed that age < 60 or ≥ 60 years old (*P* < 0.001), Stage 0+I+II or Stage III+IV (*P* < 0.001), and Breslow′s depth < 3 or ≥ 3 cm (*P* < 0.001) could distinguish the patients to favourable and poor outcome subgroups; however, the gender could not (*P* = 0.34) (Figure 2(b)).

### 3.3. The Establishment and Validation of FCRL-Determined Prognostic Signature

**Figure 3:**
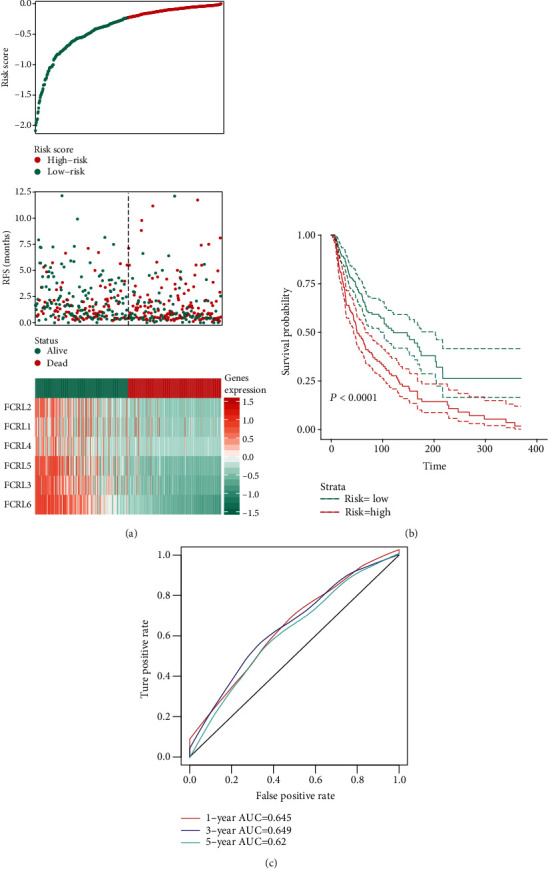
Establishment of FCRL OS prediction value in the TCGA-CM cohort. (a) Risk score for each patient in TCGA-CM cohort and heatmap to show the expression of FCRL1-6. (b) The K-M plot showing the different OS outcome in high-risk and low-risk groups. (c) ROC curve showing the AUC value in 1-year, 3-year, and 5-year predicted accuracy.

The KIRC-CM cohort was used to establish the predicted signature, based on the results of multivariate Cox Regression analysis, the risk score formula risk score = 0.276∗FCRL1_exp_ − 0.356∗FCRL2_exp_ − 0.097∗FCRL3_exp_ + 0.5∗FCRL4_exp_ − 0.208∗FCRL5_exp_ − 0.453∗FCRL6_exp_ was used to calculate the risk of each patients. FCRLA and FCRLB were not involved in the signature because they were not associated with the OS (Figure 2(a)). The risk of patients and mRNA expression of FCRLs in the TCGA-CM cohort are shown in Figure 3(a). The K-M plot indicated that the patients with high risk suffered from a poor OS outcome (*P* < 0.001, Figure 3(b)), and the AUC value of the ROC curve is meaningful (1 − year AUC = 0.645, 3 − year AUC = 0.649, and 5 − year AUC = 0.620, Figure 3(c)).

**Figure 4:**
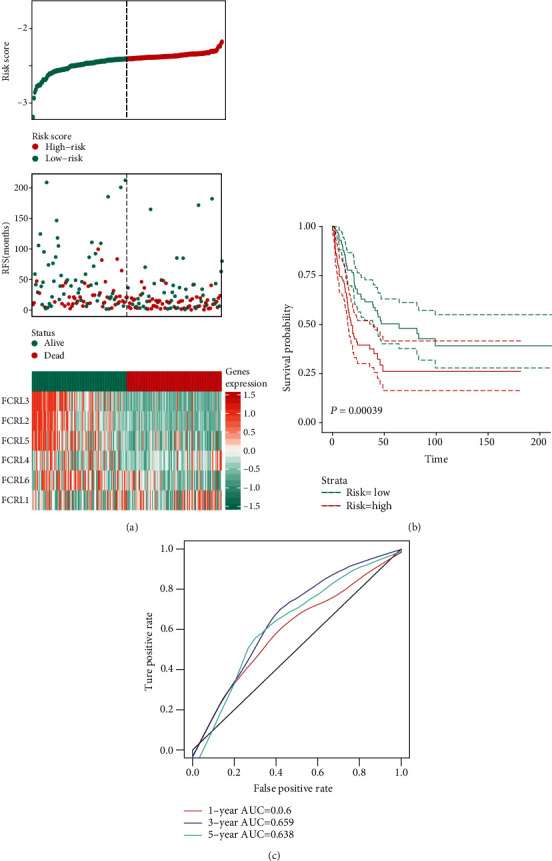
Validation of FCRL OS prediction value in the GSE65904 cohort. (a) Risk score for each patient in GSE65904 cohort and heatmap to show the expression of FCRL1-6. (b) The K-M plot showing the different OS outcome in high-risk and low-risk groups. (c) ROC curve showing the AUC value in 1-year, 3-year, and 5-year predicted accuracy.

The predicted value of FCRL-determined signatures was also validated in the GSE65904 cohort. All the patients were firstly calculated with the risk score (Figure 4(a)). And the K-M survival plot revealed that patients with the high risk show a poor prognosis as compared with the low-risk patients (*P* < 0.001) (Figure 4(b)). The AUC value of the ROC curve is meaningful (1 − year AUC = 0.600, 3 − year AUC = 0.659, and 5 − year AUC = 0.638, Figure 4(c)).

### 3.4. GSEA Analysis to Expose the FCRL-Affected Signaling Pathways

**Figure 5:**
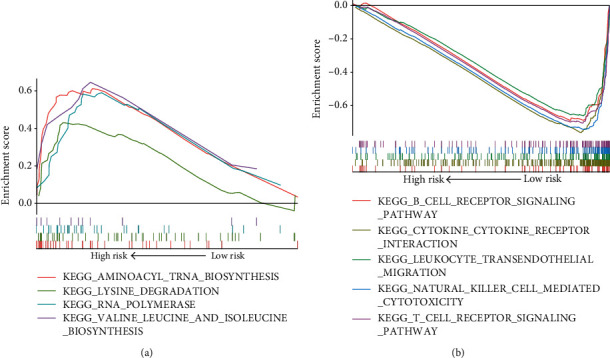
GSEA results showing the highly activated pathways in FCRL-determined high-risk and low-risk groups. (a) The activated signaling pathways in FCRL-determined high-risk group. (b) The activated signaling pathways in FCRL-determined low-risk group.

To reveal the mechanism of how FCRLs affected the process of CM, we performed the GSEA analysis to generate the potential signaling pathways. The analysis was completed between the high- and low-risk patients in the TCGA-CM cohort. Significantly, we found RNA polymerase and acid metabolism pathways to be activated in the high-risk group **(**Figure 5(a), Table 1), while in the low-risk group, which means high-expressed FCRLs, the following immune-associated pathways were activated: natural killer cell- (NK-) mediated cytotoxicity, leukocyte transendothelial migration, T cell receptor signaling pathway, and B cell receptor signaling pathway (Figure 5(b), Table 1).

### 3.5. FCRLs Affect the Progress of CM through B Cells

**Figure 6:**
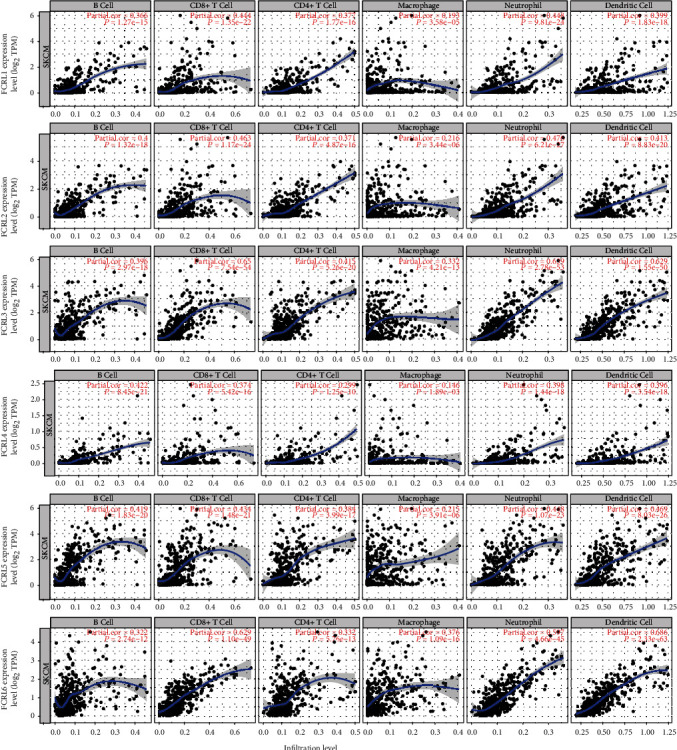
The association between FCRL1-6 and infiltration of tumor purity, B cells, CD8+ cells, CD4+ cells, macrophage, neutrophils, and dendritic cells. The FCRL1-6 expression level was displayed by the log_2_ RSEM value; Pearson's correlation analysis was used to assess the correlations. *P* < 0.05 was considered significant difference.

**Figure 7:**
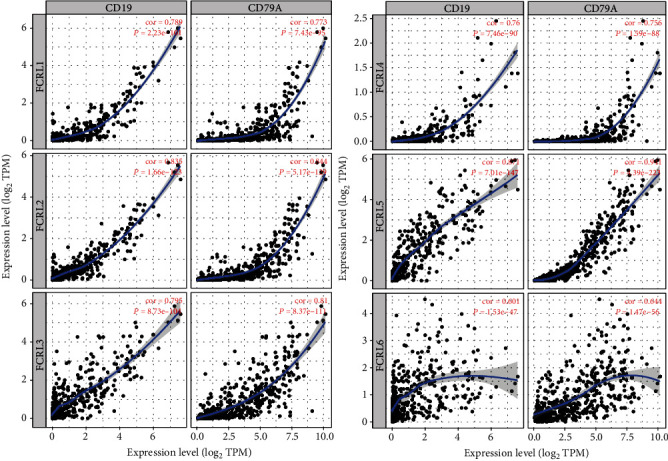
The association between FCRL1-6 and B cell markers, CD19 and CD79A. The FCRL1-6 expression level was displayed by the log_2_ RSEM value; Pearson's correlation analysis was used to assess the correlations. *P* < 0.05 was considered significant difference.

Based on the results of GSEA, we evaluated the connection between FCRLs and immune cells with TIMER. As shown in Figure 6, we can see that all the FCRL1-6 were significantly positively linked with B cells, CD8+ T cells, CD4+ T cells, neutrophils, and dendritic cells (all, *P* < 0.05), while not being significantly positively linked with macrophages. The association between FCRL1-6 and B cells is the most significant, and all the *R*-values are almost higher than 0.3, so we also analyzed the association between FCRLs and two marker genes of B cell, CD19 and CD79A. As shown in Figure 7, all the FCRL1-6 are both highly correlated with CD19 and CD79A among all the CM patients (all, *P* < 0.05, *R* > 0.6).

## 4. Discussion

CM is a common immune-related malignancy in the West, causing the majority (75%) of deaths related to skin cancer [8, 28]. Many kinds of research have revealed that an intact immune system can prevent/control and shape/promote cancer [29, 30]. The effects of FCRLs on the immune system have been discovered in some autoimmune disease, such as Graves' disease [21], systemic lupus erythematosus [23], and rheumatoid arthritis [31]. Rostamzadeh et al. [32] also demonstrated that FCRL overexpression in B cell malignancies introduce them as logical candidates for the development of antibody- and cell-based immunotherapy approaches in B cell malignancies and immune-mediated and infectious diseases. The relationship between immune system response and CM may be achieved through FCRL2 and FCRLA [24, 33]; however, the overall prognostic value of FCRLs on the OS of CM has not been clearly demonstrated yet.

Our study hypothesized that the expression of FCRLs affects the outcome of CM. And the results associated with Breslow's depth revealed the expression of FCRL1-6 was lower in patients with a Breslow's depth of 3 cm or greater. Further results showed that the lower expression of FCRL1-6 all depicted the poor OS. Furthermore, as we evaluated the prognostic value of pathological and clinical features, we found that OS was worse in patients older than 60-year-old and pathological Stages III and IV. The overall prognostic value of FCRLs was confirmed in the TCGA-CM cohort and GSE65904 cohort. Based on our findings, we can conclude that the expression of FCRL1-6 is beneficial to predict the prognosis of CM patients.

To study the potential mechanism of how FCRLs affect the OS of CM patients, we conducted the GSEA analysis among the FCRL-determined high- and low-risk groups. We found that in the low-risk group, several immune-related pathways were activated, including NK-mediated cytotoxicity, leukocyte transendothelial migration, T cell receptor signaling pathway, and B cell receptor signaling pathway. These activated pathways might act as the protect factors and associated with the favourable outcome of CM. We found some studies to prove the new findings. Baginska et al. [34] revealed that Beclin 1 could suppress the progression of tumors through facilitating NK-mediated tumor cell killing in breast and melanoma tumor in mice model. Therefore, we analyzed the association between FCRLs and immune cell infiltration in CM and found that all the FCRL1-6 are significantly associated with the infiltration rate of B cells, and the further steps confirmed these findings; the expression of FCRL1-6 is correlated with the expression of B cell markers, CD19 and CD79A. The result is consistent with the results of the study by Li et al. They demonstrate that FCRL3 has a potent costimulatory effect on Toll-Like Receptor 9- (TLR9-) mediated B cell activation but inhibits plasma cell differentiation as well [35]. Meanwhile, the association between B cells and CM was also be widely reported. Saul et al. [36] demonstrated that, in patients with metastatic CM, tumor-infiltrating B cells had increased B cell receptor class switching and affinity maturation, which means the presence of an active antigen-driven B cell response. Gilbert et al. reported that patient-derived B cells can kill melanoma cells in vitro by antibody-dependent cytotoxicity (ADCC) [37].

Based on our findings, some of the advantages of our research are worth highlighting. First and foremost, this is the first study to comprehensively describe the relationship between FCRLs and CM and analyze the prognostic value of several pathological and clinical features, such as ages, Breslow's depth, and tumor stages. Secondly, we used the TCGA-CM data to establish the predicted signature of these FCRL1-6 and validate the predict value of it in the GSE65904 cohort. Thirdly, the potential signaling pathways impacted by FCRLs were also exposed, and the NK-mediated cytotoxicity, leukocyte transendothelial migration, T cell receptor, and B cell receptor pathways might be the key FCRL-affected pathways which might benefit the outcome of CM patients, as well as the infiltration of B cells. All in all, based on the information presented in the current study, FCRLs can be used as biomarkers to predict the risk of disease progression and the OS for CM patients. Our results could help the clinical prognosis evaluation and help to choose the most adaptive treatment according to the expression level of FCRLs. On the other hand, we should also clearly recognize that our research has some limitations. Firstly, the effects of FCRLA and FCRLB on CM need to be further studied to obtain more comprehensive and accurate conclusions. Secondly, we could determine only how the FCRLs affected CM based on limited clinical data, such as tumor stage and OS, but we lack in-depth research on the impact of the environment, lifestyle, and other risk factors, so future research should consider other genetic-behaviors, gene-gene interactions to ensure a better and more comprehensive understanding of the relationship between the FCRLs and the risk of CM.

## 5. Conclusion

In conclusion, our study revealed that highly expressed FCRLs were associated with favourable OS in CM, with the exception of FCRLA and FCRLB. FCRL1-6-based prediction signature was established in TCGA-CM database and validated in GSE65904 and could act as a biomarker to predict the prognosis of patients with CM.

## Figures and Tables

**Table 1:** The results of GSEA analysis in high- and low-risk groups determined by FCRLs.

Pathway	Size	NES	NOM *P* value
High-risk group			
KEGG_GLYCOSYLPHOSPHATIDYLINOSITOL_GPI_ANCHOR_BIOSYNTHESIS	25	1.997	<0.001
KEGG_RNA_POLYMERASE	29	1.849	0.008
KEGG_AMINOACYL_TRNA_BIOSYNTHESIS	41	1.848	0.008
KEGG_GLYOXYLATE_AND_DICARBOXYLATE_METABOLISM	16	1.726	0.014
KEGG_VALINE_LEUCINE_AND_ISOLEUCINE_BIOSYNTHESIS	11	1.611	0.03
KEGG_LYSINE_DEGRADATION	44	1.526	0.05
Low-risk group			
KEGG_CYTOKINE_CYTOKINE_RECEPTOR_INTERACTION	258	-2.777	<0.001
KEGG_CHEMOKINE_SIGNALING_PATHWAY	186	-2.765	<0.001
KEGG_CELL_ADHESION_MOLECULES_CAMS	131	-2.716	<0.001
KEGG_NATURAL_KILLER_CELL_MEDIATED_CYTOTOXICITY	130	-2.694	<0.001
KEGG_VIRAL_MYOCARDITIS	68	-2.647	<0.001
KEGG_JAK_STAT_SIGNALING_PATHWAY	151	-2.634	<0.001
KEGG_ANTIGEN_PROCESSING_AND_PRESENTATION	79	-2.578	<0.001
KEGG_LEUKOCYTE_TRANSENDOTHELIAL_MIGRATION	116	-2.565	<0.001
KEGG_TOLL_LIKE_RECEPTOR_SIGNALING_PATHWAY	101	-2.525	<0.001
KEGG_LEISHMANIA_INFECTION	69	-2.483	<0.001
KEGG_T_CELL_RECEPTOR_SIGNALING_PATHWAY	107	-2.478	<0.001
KEGG_SYSTEMIC_LUPUS_ERYTHEMATOSUS	131	-2.477	<0.001
KEGG_FC_GAMMA_R_MEDIATED_PHAGOCYTOSIS	95	-2.468	<0.001
KEGG_HEMATOPOIETIC_CELL_LINEAGE	85	-2.458	<0.001
KEGG_NOD_LIKE_RECEPTOR_SIGNALING_PATHWAY	61	-2.414	<0.001
KEGG_AUTOIMMUNE_THYROID_DISEASE	50	-2.39	<0.001
KEGG_APOPTOSIS	86	-2.34	<0.001
KEGG_B_CELL_RECEPTOR_SIGNALING_PATHWAY	74	-2.318	<0.001
KEGG_FC_EPSILON_RI_SIGNALING_PATHWAY	79	-2.304	<0.001

## Data Availability

The data used to support the findings of this study are included within the article. The gene expression data can be accessed on The Cancer Genome Atlas (TCGA) and Gene Expression Omnibus (GEO).
